# Multiple Primary Angiosarcomas of the Colon

**DOI:** 10.1155/2021/7237379

**Published:** 2021-09-09

**Authors:** Sonja Radić, Mario Zovak, Anita Galović Marić, Stjepan Baturina, Monica Stephany Kirigin, Božo Krušlin

**Affiliations:** ^1^Department of Pathology and Cytology, General Hospital Karlovac, Karlovac, Croatia; ^2^Department of Surgery, Sestre Milosrdnice University Hospital Center, Zagreb, Croatia; ^3^Department of Radiology, Sestre Milosrdnice University Hospital Center, Zagreb, Croatia; ^4^Ljudevit Jurak Department of Pathology and Cytology, Sestre Milosrdnice University Hospital Center, Zagreb, Croatia

## Abstract

**Introduction:**

Gastrointestinal angiosarcomas are rare and represent less than 1% of all gastrointestinal tract malignancies, with most occurring in the stomach and small intestine. Occurrence in the colorectal segments is considered extremely rare. *Case Report*. We describe the case of a 61-year-old male with multiple primary angiosarcomas of the colon who presented with fever and abdominal pain. The patient was initially hospitalized and treated as having an infectious disease. A multislice computed tomography (MSCT) scan revealed multiple soft tissue tumors in the region of the left iliopsoas and gluteus medius muscles. After developing hematochezia, a colonoscopy was performed which found an ulcerated tumor in the sigmoid colon. The small tissue biopsy taken during the procedure presented diagnostic difficulties and was given a preliminary diagnosis of gastrointestinal stromal tumor (GIST). Examination of the resected colon segment and surrounding fat tissue revealed four separate tumors. Microscopically, the tumors were composed of solid sheets of spindle and epithelioid neoplastic cells with prominent nucleoli and numerous mitotic figures and immunohistochemically positive for ERG, CD31, CD34, vimentin, and CD117, while negative for CK7, CK20, CD20, CD3, CD45, TTF-1, PAN-CK, ALK, Mpox, S-100, and DOG1, leading to the final diagnosis of multiple colonic angiosarcomas. The patient's condition declined rapidly and he passed away from multiple organ failures 60 days after initial hospitalization.

**Conclusion:**

Both clinical and pathological diagnoses of colorectal angiosarcoma are challenging. Patients are present with nonspecific symptoms leading to mismanagement and late diagnosis. A definitive pathological diagnosis relies on immunohistochemical staining for endothelial markers. Misdiagnosis as poorly differentiated adenocarcinoma or GIST is possible in limited tissue biopsies.

## 1. Introduction

Angiosarcomas are rare malignant tumors that arise from the vascular endothelium of lymphatic or blood vessels. They account for less than 1% of all soft tissue sarcomas and occur most commonly in the skin and subcutaneous soft tissue, and less commonly in the breast, liver, bone, and spleen. Gastrointestinal angiosarcomas are rare and represent less than 1% of all gastrointestinal tract malignancies, with most occurring in the stomach and small intestine [1–3]. Occurrence in the colorectal region is considered extremely rare, with less than 35 cases reported in the literature so far [4]. Colorectal angiosarcoma may be easily misdiagnosed as poorly differentiated adenocarcinoma or gastrointestinal stromal tumor (GIST) in limited tissue biopsies [5].

We describe the case of a 61-year-old male with multiple primary angiosarcomas of the colon who presented with fever, abdominal pain, and hematochezia.

## 2. Case Report

A 61-year-old male was admitted to the Clinic for Infectious Diseases “Dr. Fran Mihaljevic”, Zagreb, Croatia, in July 2019, presenting with a fever of 39°C, accompanied with night sweats, vomiting, and pain in the lower left abdomen during a period of one month. Past medical history included arterial hypertension controlled by amlodipine. Routine blood tests showed elevated inflammatory markers (CRP 136-165 mg/L, SE 100 mm/h, leucocytes 8.7 × 10^9^/L). The patient was treated with multiple antibiotics (azithromycin, clarithromycin, ceftriaxone, cloxacillin, ciprofloxacin, vancomycin, and meropenem) and tested negative for a number of infectious diseases (hemorrhagic fever with renal syndrome, Q fever, brucellosis, Bartonella, and tuberculosis). Tests for tumor markers (CEA, CA19-9, AFP, and PSA) were negative. A multislice computed tomography (MSCT) scan with contrast of the thorax, abdomen, and pelvis revealed multiple expansive, hypovascularized soft tissue tumors up to 4 cm in diameter in the region of the left iliopsoas and gluteus medius muscles.

**Figure 1:**
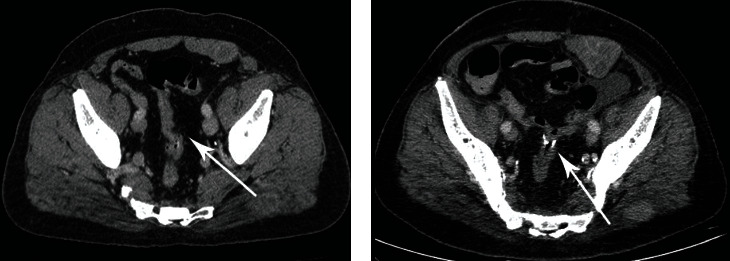
(a) The preoperative CT scan shows a stricture of the sigmoid colon. (b) A resection of the sigmoid colon was performed and the segment sent for histological analysis.

17 days after initial hospitalization, the patient presented with hematochezia and was subsequently transferred to the Sestre Milosrdnice University Hospital Center, Zagreb, Croatia, where a colonoscopy was performed, which found an ulcerated, hemorrhagic subepithelial tumor in the sigmoid colon. Specimens of the lesion were taken for microscopic examination. Resection of the rectosigmoid colon with colorectal anastomosis was performed (Figures 1(a) and 1(b)).

H&E stained slides of the sample taken during colonoscopy revealed polymorphic, poorly differentiated tumor cells. Immunohistochemistry found the tumor cells to be positive for CD117, SMA, and S-100, while negative for CD34, chromogranin A, CK7, CK20, PAN-CK, HMB45, synaptophysin, and PAX-8. A preliminary diagnosis of poorly differentiated gastrointestinal stromal tumor (GIST) was given.

Gross pathological examination of the resected colon segment and surrounding fat tissue revealed four separate tumors. One tumor was the ulcerated lesion seen and biopsied during colonoscopy, measuring 2 cm in greatest diameter, while the remaining three were hemorrhagic nodules ranging from 0.6 cm to 2 cm in diameter, located in the surrounding fat tissue.

**Figure 2:**
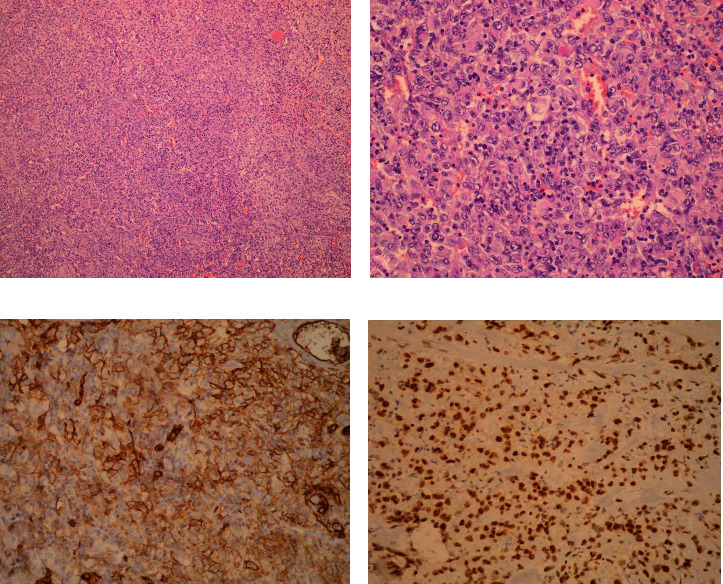
(a) All four tumors from the resected colon segment were composed of solid sheets of spindle and epithelioid neoplastic cells (H&E, 100x magnification). (b) At higher magnification, prominent nucleoli and mitotic figures are visible (H&E, 400x magnification). (c) The tumor cells showed strong positive immunohistochemical staining with the endothelial marker CD31 (200x magnification). (d) Staining for ERG confirmed the tumors' endothelial origin (200x magnification).

Microscopically, H&E sections of all tumors showed seemingly solid sheets of spindle and epithelioid neoplastic cells with irregular nuclei, prominent nucleoli, and numerous mitotic figures. At higher magnification, fissures between the neoplastic cells containing erythrocytes could be discerned. Immunohistochemical analysis performed on samples of the largest nodular tumor revealed that the neoplastic cells were positive for ERG, CD31, CD34, vimentin, and CD117, while negative for CK7, CK20, CD20, CD3, CD45, TTF-1, PAN-CK, ALK, Mpox, S-100, and DOG1 (Figures 2(a)–2(d)). The Ki-67 index was over 90%, indicating exceedingly high proliferative activity. The final pathological diagnosis was that of multiple primary colonic angiosarcomas.

**Figure 3:**
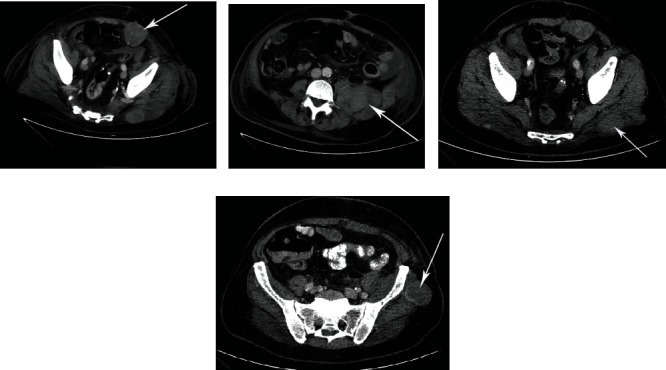
A CT scan following the surgery showed multiple residual tumors in the lower abdomen and pelvis. (a) Front abdominal wall. (b) Psoas muscle. (c) Gluteus. (d) Iliac bone.

The patient's overall condition declined significantly following surgery. A CT scan showed multiple residual tumors in the lower abdomen and pelvis measuring up to 6.5 cm in the greatest diameter, with signs of massive hemorrhage within them (Figures 3(a)–3(d)). The patient passed away from multiple organ failures in September 2019, 60 days after initial hospitalization. An autopsy was not performed.

## 3. Discussion

To our knowledge, 33 cases of primary colorectal angiosarcoma have been reported in the literature [4]. The first report was given by Steiner and Palmer in 1949 [6]. Only 9 of these reports were made prior to 2004; as of 2004, new cases have been described on almost a yearly basis, making use of immunohistochemical staining primarily for endothelial markers CD31 and CD34 [1–21]. In view of colorectal angiosarcoma being easily misdiagnosed as poorly differentiated adenocarcinoma or gastrointestinal stromal tumor (GIST) in limited tissue biopsies [5], the actual occurrence of angiosarcoma in the colorectal region may be less rare than previously thought.

The CD117 positivity of the tumor cells in our case presented an additional diagnostic difficulty. CD117 is routinely used as a marker of GIST, which shows a positivity rate of 80-100%. Two additional immunohistochemical markers may be used to differentiate between GIST and other morphologically similar tumors—DOG1 and PKC*θ*. DOG1 positivity is expressed in 57-96% of GISTs and directly correlated with CD117 positivity, with CD117-positive GISTs being also DOG1-positive. However, DOG1 does not usually mark other tumors and was negative in the present case, ultimately eliminating the suspicion of GIST. Protein kinase C theta (PKC*θ*) is positive in 72-100% of GISTs, but not in other CD117-positive non-GIST soft tissue sarcomas and other tumors, and has diagnostic value in the uncommon double CD117/DOG1-negative GISTs [22].

Inflammatory myofibroblastic tumors (IMT) microscopically present with similar features, consisting of spindled to epithelioid myofibroblasts with admixed inflammatory cells, predominantly of mononuclear type, and may also be misdiagnosed as GIST based on morphology. However, the tumor cells of IMT are characteristically positive for vimentin and negative for CD117 and CD34, as well as frequently showing ALK positivity [23], an immunohistochemical profile not consistent with our case.

Microscopically, angiosarcoma may potentially be misdiagnosed as a poorly differentiated carcinoma. The immunophenotype of negative CK7 and positive CK20 is considered a characteristic of colorectal adenocarcinomas. However, a CK20-negative phenotype was found to be associated with microsatellite instability and BRAF mutation [24]. In our case, the tumors were negative for CK7, CK20, and PAN-CK.

Kaposi's sarcoma (KS) is not an uncommon disease among patients with acquired immunodeficiency syndrome (AIDS), classically presenting as a spindle cell proliferation with irregular vascular channels and extensive red blood cell extravasation. Histological features of KS may overlap with angiosarcoma, GIST, and spindle cell melanoma. Immunohistochemically, KS shows positivity for CD34 and CD117, but is distinguished from other tumors by positive staining for human herpes virus 8 latent nuclear antigen (HHV8 LNA) [25].

Gastrointestinal melanomas are relatively rare and frequently metastasize from cutaneous primaries. These tumors may contain obvious cytoplasmic melanin granules aiding identification and differentiation from other spindle cell lesions. However, a definitive diagnosis for amelanotic cases depends on positive immunohistochemical stains for S-100, HMB45, and vimentin [26].

Several predisposing factors for the development of angiosarcoma have been suggested, including radiation therapy, chronic lymphedema, long-term exposure to arsenic, vinyl chloride, thorium oxide, and other chemicals, as well as foreign objects inside the body such as unremoved surgical material [4, 7, 8]. Our patient had no known exposure to any of these factors. Although a direct relationship has not been established, angiosarcoma has also been reported in patients who take calcium channel blockers [4], and our patient was known to be taking amlodipine.

The sigmoid colon has been reported as the most common site of involvement, followed by the rectum. The size of reported tumors ranged from 1.5 to 12 cm [1–5, 9]. The main clinical manifestation of colorectal angiosarcoma in the published reports was gastrointestinal bleeding, occurring in 67% of the cases, with abdominal pain in second place with 46% of cases. Fever, which was our patient's primary symptom and cause for initial hospitalization, was described in 3% of the cases.

Histological type was reported as epithelioid in 52%, spindle in 29%, and mixed epithelioid and spindle cell as seen in our patient in 19% of the cases. In the cases where immunohistochemical findings were reported, 86% of tumors were positive for CD31, and 80% were positive for CD34 [4]. Angiosarcomas may be positive for both CD34 and CD117, generally regarded as markers for GIST [4, 5].

The general prognosis for colorectal carcinoma is very poor, with late diagnosis due to patients presenting with nonspecific symptoms, and many cases developing systemic metastasis within a year of diagnosis. Complete surgical resection of the tumor remains the most effective form of treatment.

## 4. Conclusion

Colorectal angiosarcoma is a rare and aggressive malignancy that clinically presents with nonspecific symptoms, leading to late diagnosis. A definitive pathological diagnosis relies on immunohistochemical staining for endothelial markers. Misdiagnosis as poorly differentiated adenocarcinoma or GIST is possible in limited tissue biopsies.

## Data Availability

The original reports are available from the corresponding author upon request.
